# Aquaporin-Mediated Water and Hydrogen Peroxide Transport Is Involved in Normal Human Spermatozoa Functioning

**DOI:** 10.3390/ijms18010066

**Published:** 2016-12-30

**Authors:** Umberto Laforenza, Giorgia Pellavio, Anna Lisa Marchetti, Claudia Omes, Federica Todaro, Giulia Gastaldi

**Affiliations:** 1 Department of Molecular Medicine, University of Pavia, I-27100 Pavia, Italy; giorgia.pellavio@gmail.com (G.P.); gastaldi@unipv.it (G.G.); 2 Scientific Direction, Fondazione IRCCS Policlinico San Matteo, I-27100 Pavia, Italy; a.marchetti@smatteo.pv.it; 3 Center for Reproductive Medicine, Obstetrics and Ginecology Unit, Fondazione IRCCS Policlinico San Matteo, I-27100 Pavia, Italy; claudia.omes@unipv.it (C.O.); todarofederica@gmail.com (F.T.)

**Keywords:** water channel, oxidative stress, sterility, aquaporins-8, aquaporins-7, sperm motility

## Abstract

Different aquaporins (AQPs) are expressed in human sperm cells and with a different localization. Their function has been related to cell volume control in response to the osmotic changes encountered passing from the epididymal fluid to the cervical mucus or involved in the end stage of cytoplasm removal during sperm maturation. Recently, AQPs have also shown hydrogen peroxide (H_2_O_2_) permeability properties. Here, we investigate the expression, localization and functioning of AQPs in human sperm cells with particular attention to their role as peroxiporins in reactive oxygen species (ROS) scavenging in both normospermic and sub-fertile human subjects. Western blotting and immunocytochemistry were used to confirm and clarify the AQPs expression and localization. Water and H_2_O_2_ permeability was tested by stopped flow light scattering method and by the CM-H2DCFDA (5-(and-6)-chloromethyl-2′,7′-dichlorodihydro-fluorescein diacetate, acetyl ester) H_2_O_2_ fluorescence probe, respectively. AQP3, -7, -8, and -11 proteins were found in human sperm cells and localized in the head (AQP7), in the middle piece (AQP8) and in the tail (AQP3 and -11) in both the plasma membrane and in intracellular structures. Sperm cells showed water and H_2_O_2_ permeability which was reversibly inhibited by H_2_O_2_, heat stress and the AQP inhibitor HgCl_2_. Reduced functionality was observed in patients with compromised basal semen parameters. Present findings suggest that AQPs are involved in both volume regulation and ROS elimination. The relationship between sperm number and motility and AQP functioning was also demonstrated.

## 1. Introduction

The major physiological determinants of male fertility are sperm motility, number and morphology. Among the many factors that may compromise fertility, oxidative stress is believed to play an important role [1]. In fact, elevated levels of reactive oxygen species (ROS) were found in the seminal plasma in 30%–40% of infertile subjects [2]. In all cells, ROS can have a different effect depending on their concentration: a physiological effect at low concentrations, acting as signaling molecules (second messengers) and a cytotoxic effect at high concentrations. High levels of ROS determine negative effects in spermatozoa such as decreased viability, decreased motility and increased mid-piece sperm morphological defects [3,4]. On the contrary, low ROS levels are involved in normal physiological functions like acrosome reaction, sperm hyperactivation and capacitation, sperm fertilizing potential and mitochondria functioning in the mid-piece that generates energy for the flagellar beat and thus sperm motility [5,6,7].

Overall, the oxidative stress derives from the imbalance between ROS production and scavenging. A large number of ROS are generated in mammalian spermatozoa and the most abundant is the superoxide anion, O_2_^−^, that is rapidly converted by superoxide dismutase (SOD) to hydrogen peroxide, H_2_O_2_ [1,8,9]. In the ejaculate, ROS are generated by leukocytes, immature, mature and dead spermatozoa, and by some pathological conditions such as varicocele [10,11,12,13,14,15,16]. Moreover, other factors and conditions can generate ROS: smoking, alcohol consumption, xenobiotics, toxic metals, heat, mobile phone radiation, age and obesity (for a comprehensive overview see [1,17]). Many pathways can generate ROS, and thus H_2_O_2_ in human spermatozoa: (1) NADPH oxidase in the plasma membrane; (2) NADH dependent oxido-reductase in the mitochondria; and (3) NADPH oxidase 5 in the equatorial and post-acrosomal regions [18,19].

As concerns ROS scavenging, the ejaculate possesses different detoxification pathways: chemical antioxidants (vitamin C and E, zinc and selenium, l-carnitine and coenzyme Q10, carotenoids, cysteine, and albumin) present in the seminal plasma, and enzymatic antioxidant systems (SOD, catalase and glutathione peroxidase), as reviewed by [2,9,17]. Recently, the diffusion of H_2_O_2_ from the producing cells across the plasma membranes to the extracellular fluid has been also considered an important ROS scavenging possibility [20,21]. In the past, it was thought that hydrogen peroxide could freely diffuse across biological membranes but successively this has been questioned [22]. Both the striking physical-chemical similarities between water and hydrogen peroxide, and the capacity of some aquaporins (AQPs) to be permeated by glycerol, urea and other small solutes in addition to water, suggested a role of AQPs as peroxiporins [21].

AQPs are a family of water channel proteins present in mammals in different isoforms (thirteen to date, named from AQP0–12) with different permeability characteristics and different cellular and subcellular localizations [23]. Some of them were found to facilitate the diffusion of H_2_O_2_: AQP3, -8, and -9 [24,25,26,27,28]. To date, four AQPs were found in human spermatozoa: AQP3, -7, -8 and -11 [29].

AQP3 is localized in the principal piece of the sperm tail membrane. In AQP3 null mice, sperm cells show normal motility but an impaired migration capacity into the oviduct, resulting in a reduced male fertility [29].

AQP7 has been identified by immunofluorescence and fluorescence-activated cell sorter (FACS) in human sperm cells and is localized at the middle piece and the anterior tail portion [30]. Moreover, some infertile patients lacking AQP7 expression have lower sperm motility. Other authors found an intense AQP7 staining in the tail of ejaculated human sperm cells [31,32].

As concerns AQP8, the labeling was found in punctuate cytoplasmic droplets and in the tail of ejaculated spermatozoa [31,32]. Functionally, sperm AQP7 expression has been correlated with progressive motility (PR) and is reduced in patients, while an inverse correlation has been found between AQP8 expression and the sperm coiling entity [32].

Finally, AQP11, which is characterized by an exclusively intracellular localization, was also found in human spermatozoa at mRNA level [33]. Immunocytochemistry of AQP11 revealed an intense staining on the distal quarter of the sperm tail in mice and rats [31,33] but this has not yet been proven in humans.

Recently, in the marine teleost *Sparus aurata*, a direct involvement of mitochondrial Aqp8b-mediated H_2_O_2_ efflux (scavenging) was observed, which allows detoxification and the preservation of spermatozoa motility [34].

In this study we examined in human ejaculated sperm cells from normospermic and sub-fertile subjects: (1) the expression of AQP3, -7, -8 and -11 protein by immunoblotting; (2) the cellular localization of the AQPs by immunocytochemistry; and (3) water and H_2_O_2_ permeability and their features, measured by a stopped-flow light scattering method and using an H_2_O_2_-selective fluorescent probe (5-(and-6)-chloromethyl-2′,7′-dichlorodihydrofluorescein diacetate, acetyl ester; CM-H2DCFDA), respectively.

The results here reported provide evidence that AQPs have a fundamental role in regulating sperm cell volume and in maintaining their motility permitting ROS scavenging.

## 2. Results

### 2.1. Semen Characteristics in Normospermic and Sub-Fertile Subjects

Table 1 lists semen characteristics of all subjects either normospermic (*n* = 53) and sub-fertile (*n* = 26) included in the study, who attended the clinic because of infertility. Although the volume of ejaculate was not statistically different in the two groups of subjects examined, as expected both sperm count and progressive motility were dramatically reduced in sub-fertile patients, by about 75% the former and 58% the latter. Motile sperm count was significantly reduced in sub-fertile men (about 90%) as were total motility (about 50%), and morphology (about 63%) while non progressive motility was similar in normospermic and sub-fertile subjects.

### 2.2. Immunoblotting of AQP3, -7, -8 and -11 Protein Expression in Human Ejaculated Semen from Normospermic Subjects

**Figure 1:**
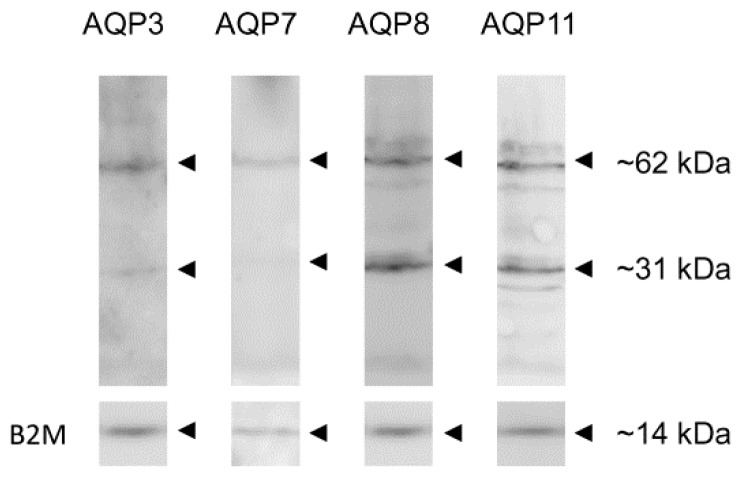
Aquaporin-3 (AQP3), -7 (AQP7), -8 (AQP8) and -11 (AQP11) protein expression in human ejaculated semen from normospermic subjects. Blots representative of three were shown. Lanes were loaded with 40 μg of proteins, probed with anti-AQP3, -7, -8 and -11 rabbit polyclonal antibodies and processed as described in Materials and Methods. The same blots were stripped and re-probed with anti-beta-2-microglobulin (B2M) polyclonal antibody, as housekeeping. Major bands of about 31 kDa (monomer) and 62 kDa (dimer) were observed.

The expression of AQP3, -7, -8, and -11 proteins in human ejaculated semen from normospermic subjects was analyzed by immunoblotting with affinity-purified antibodies. The results showed that all AQP proteins investigated were expressed (Figure 1). Immunoblots showed a major band at approximately 31 kDa, which was compatible the monomer, and a band of approximately 62 kDa, probably representing the dimer form (Figure 1, arrowheads). The specificity of the reactions was previously characterized and checked in experiments performed by incubating the blots with pre-immune rabbit serum (not shown). The expression of the housekeeping gene *B2M* was also shown (Figure 1).

### 2.3. Aquaporins Immunolocalization in Human Spermatozoa from Normospermic Subjects

**Figure 2:**
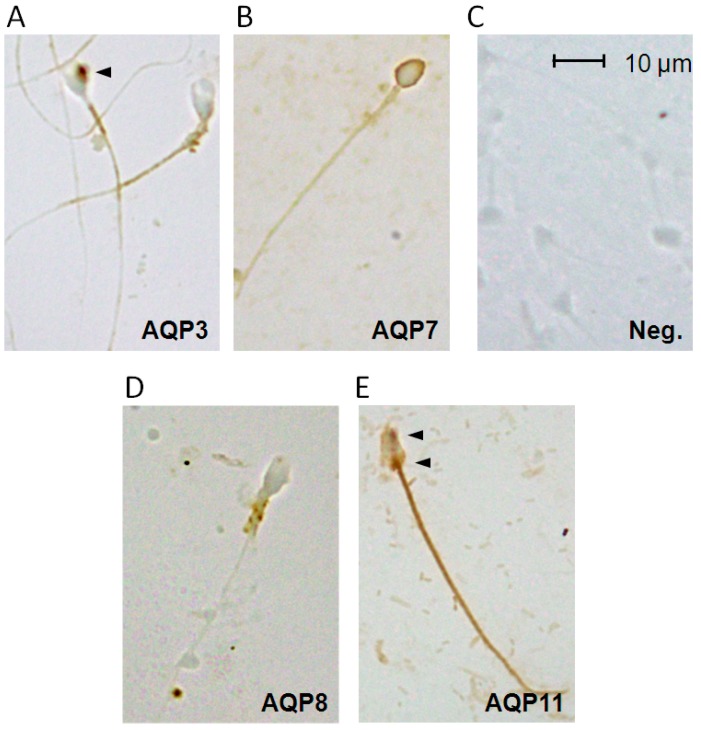
Immunocytochemical localization of the AQP3, -7, -8 and -11 proteins in human ejaculated semen from normospermic subjects: (**A**) AQP3 immunoreactivity was observed in the principal piece of the sperm tail membrane and in 3% of sperms in granules present in the head and in the midpiece (arrowhead); (**B**) intense AQP7 staining was observed in the plasma membrane region of the sperm head; (**D**) AQP8 labeled the midpiece of the spermatozoa, apparently in the mitochondria; and (**E**) AQP11 protein was localized into granules and vesicles of soma (arrowheads) and in the tail. Controls in which the primary antibody was substituted with non-immune serum show an absence of labeling (Neg.; (**C**)). Scale bar, 10 μm.

The localization of AQP3, -7, -8 and -11 proteins in human spermatozoa was investigated by immunocytochemistry. As shown in Figure 2, the anti-AQP3, -7, -8 and -11 antibodies strongly labeled different parts of human sperm cells, while negative controls (incubated with non-immune serum) gave a negligible signal (Figure 2C).

Figure 2A showed that AQP3 immunolabeling was observed in the principal piece of the sperm tail membrane and in 3% of sperms into granules present in the head and in the midpiece. AQP7 is localized in the plasma membrane region of the sperm head (Figure 2B). Interestingly, few sub-fertile and, surprisingly, few normospermic subjects did not show any AQP7 labeling (data not shown). AQP8 showed intense immunoreactivity in the midpiece of the spermatozoa, apparently in the mitochondria (Figure 2D) and in 2% of sperms in the proximity of midpiece plasma membrane (not shown). AQP11 staining was particularly evident in intracellular structures and in the tail; the labeling was observed into granules and vesicles and may represent the end stage of cytoplasm and organelle elimination process occurring during sperm maturation (Figure 2E).

### 2.4. Effect of Oxidative Stress on Water Permeability of Human Ejaculated Semen from Normospermic and Sub-Fertile Subjects

**Figure 3:**
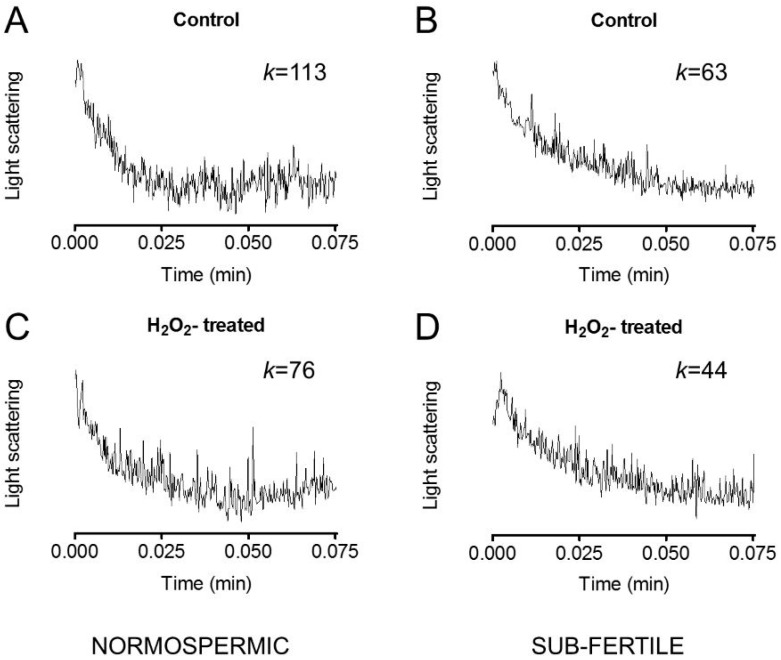
Representative traces of stopped-flow osmotic water permeability measurements obtained from human ejaculated semen of normospermic (**A**,**C**) and sub-fertile (**B**,**D**) subjects. Sperm cells were exposed to a 150 mOsm osmotic gradient in two different conditions: untreated cells (Control; (**A**,**B**)) and cells treated for 45 min with 50 μM H_2_O_2_ to induce an oxidative stress condition (H_2_O_2_-treated; (**C**,**D**)). *k* relative values of single curves are also shown.

Sperm cells exposed to a hypotonic buffer behaved as functional osmometers showing a sudden swelling (Figure 3). The decrease in scattered light intensity could be fitted by a one phase exponential decay equation and the initial rate constant *k* was obtained.

**Figure 4:**
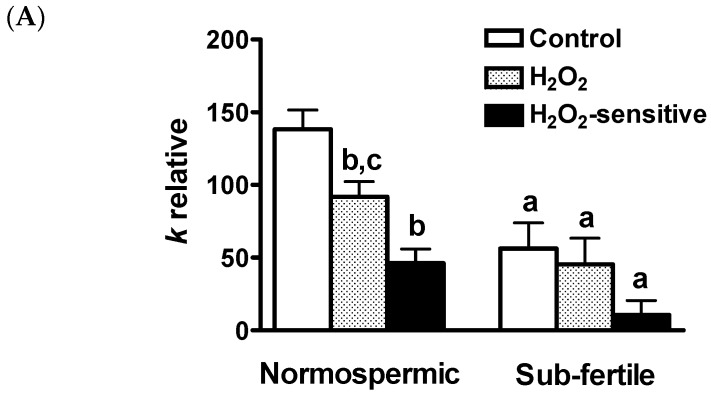

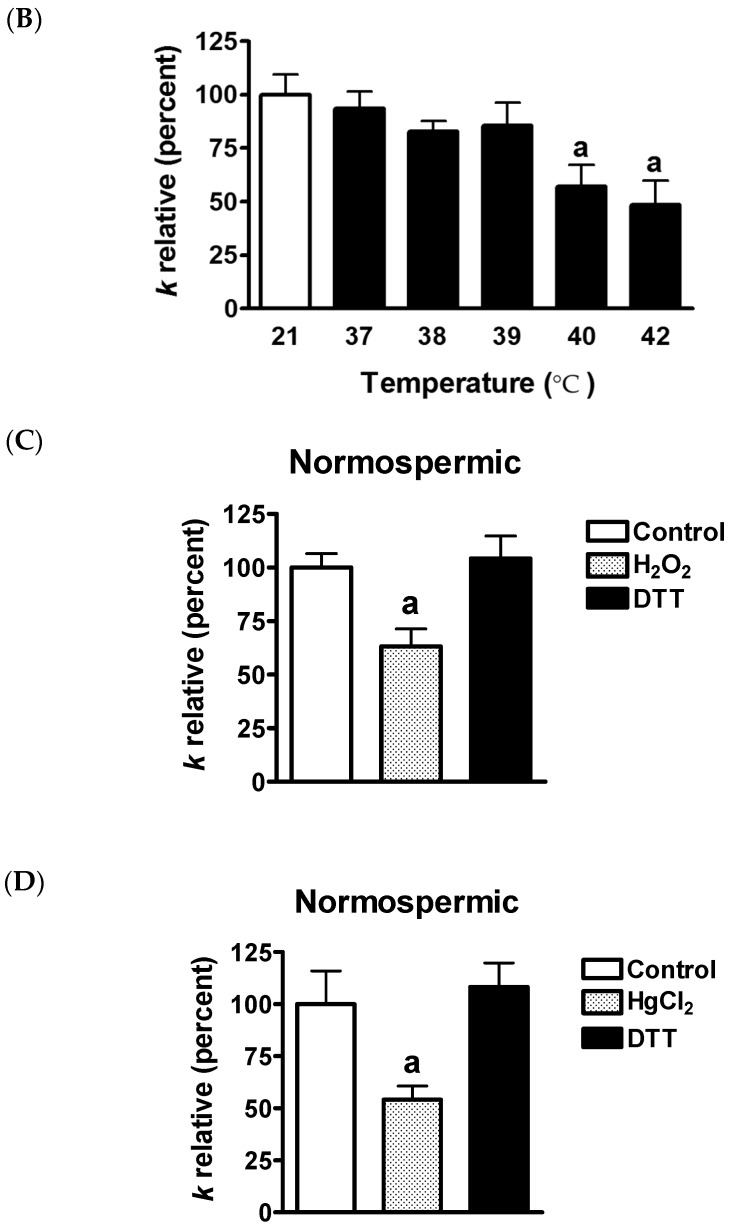
Effect of oxidative stress on the water permeability of human ejaculated semen. (**A**) Effect of hydrogen peroxide (H_2_O_2_). Sperm cells were exposed to a 150 mOsm osmotic gradient in two different conditions: untreated cells (Control) and cells treated for 45 min with 50 μM H_2_O_2_ to induce an oxidative stress condition. The H_2_O_2_-sensitive water permeability was obtained by subtracting H_2_O_2_ insensitive water permeability from total water permeability. a, *p* < 0.05 vs. normospermic (Student’s *t* test); b, *p* < 0.05 vs. Control and c, *p* < 0.05 vs. H_2_O_2_-sensitive (ANOVA, followed by Newman–Keuls’s *Q* test); (**B**) Temperature dependence: The osmotic water permeability of human ejaculated semen from normospermic subjects was measured after cells exposure for three hours to different temperature (from 21 to 42 °C). a, *p* < 0.05 vs. 21 °C (ANOVA, followed by Newman–Keuls’s *Q* test); (**C**,**D**) Reversible effect of hydrogen peroxide (H_2_O_2_) and mercury chloride (HgCl_2_). (**C**) Sperm cells were exposed to a 150 mOsm osmotic gradient in three different conditions: untreated cells (Control), cells treated for 45 min with 50 μM H_2_O_2_, cells treated with H_2_O_2_ followed by 15 min treatment with 5 mM dithiothreitol (DTT); (**D**) Sperm cells were exposed to a 150 mOsm osmotic gradient in three different conditions: normal untreated cells (Control), cells treated for 15 min with 100 μM HgCl_2_ (HgCl_2_), cells treated with HgCl_2_ followed by 15 min treatment with 5 mM DTT. a, *p* < 0.05 vs. Control and DTT (ANOVA, followed by Newman–Keuls’s *Q* test). Bars represent the osmotic water permeability of sperm cells expressed as *k* relative (**A**) or percent of *k* relative (**B**–**D**). Values are means ± SEM of 4–15 single shots for each of 8–9 different experiments.

To test whether oxidative stress was able to modify the osmotic permeability of the human sperm cells, a chemical stress was applied to the cells by adding H_2_O_2_. Figure 3 shows representative light scattering curves obtained by normospermic and sub-fertile sperm cells in normal condition and after H_2_O_2_ treatment. The water permeability in normal condition (non-stressed cells) of human ejaculated semen was three times higher in normospermic than in sub-fertile subjects (Figure 4A).

In response to exposure to H_2_O_2_ sperm cells from normospermic men showed a statistically significant reduction of water permeability of about 34%. Sub-fertile subjects, on the contrary, were unaffected by H_2_O_2_ treatment with the H_2_O_2_-sensitive water permeability almost absent (Figure 4A). The H_2_O_2_-sensitive water permeability, obtained by subtracting H_2_O_2_ insensitive water permeability from total water permeability, was also calculated: in normospermic subjects, H_2_O_2_-sensitive water permeability is similar to the total water permeability (Ctr) of sub-fertile subjects. In sub-fertile subjects, H_2_O_2_-sensitive water permeability was either very low or almost absent.

The effect of H_2_O_2_ on routine semen parameters of normorspermic and sub-fertile subjects was also evaluated and the results shown in Table 2. Normospermic patients showed a significantly decreased in progressive (PR) motility, motile sperm count, total motility (due to the decrease of PR motility) and vitality after H_2_O_2_ treatment. Sperm from sub-fertile patients have a statistically significant reduction in non progressive (NP) motility, total motility (due to the decrease of NP) and vitality, while PR motility was unchanged.

### 2.5. Temperature Dependence of the Osmotic Water Permeability of Human Ejaculated Semen from Normospermic Subjects

Heat stress is well known to negative affect the process of spermatogenesis [35], but sperm thermotaxis, a mechanism guiding spermatozoa from a cooler site to the warmer fertilization site, takes place preferentially at warmer temperatures [36,37]. To this purpose we evaluated the osmotic water permeability of human ejaculated semen from normospermic subjects exposed to different temperature (from 21 to 42 °C). Results shows that water permeability was unchanged until 40 °C but was significant reduced (about 50%) for incubation temperature of 41 and 42 °C (Figure 4B).

### 2.6. Reversible Effect of the H_2_O_2_ and HgCl_2_ Treatment on the Osmotic Water Permeability of Human Ejaculated Semen from Normospermic Subjects

The effect of oxidative stress on the osmotic permeability was further studied to assess the reversibility of H_2_O_2_ inhibitory effect. Whole human ejaculated semen from normospermic subjects were treated with a H_2_O_2_ and then with a dithiothreitol (DTT) excess. Figure 4C shows that a short DTT treatment was sufficient to restore cell permeability to water.

Involvement of AQPs in cell swelling was confirmed by the observation that the pretreatment of the sperm with the AQP water channel inhibitor Hg^2^^+^ significantly reduced about 50% of the water transport. Hg^2+^ inhibition was completely reversed by treatment with DTT (Figure 4D).

### 2.7. Effect of Mercury Chloride (HgCl_2_) Treatment on Hydrogen Peroxide Permeability and Motility of Human Ejaculated Semen from Normospermic and Sub-Fertile Subjects

**Figure 5:**
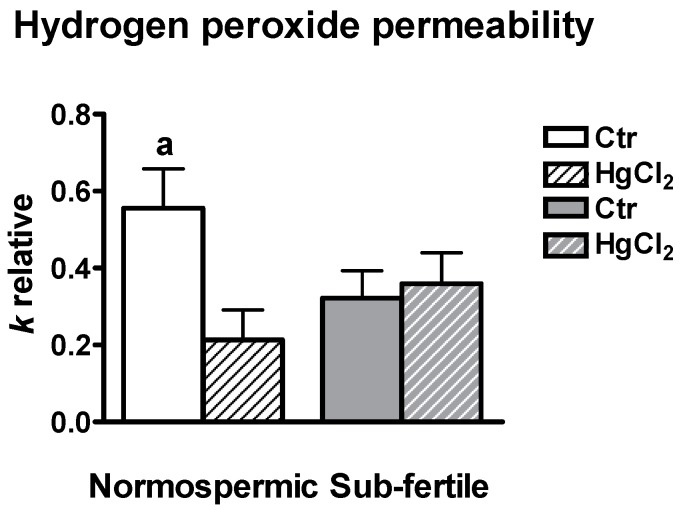
Effect of mercury chloride (HgCl_2_) treatment on the hydrogen peroxide permeability of human ejaculated semen from normospermic and sub-fertile subjects. Hydrogen peroxide permeability was measured by loading the human ejaculated semen with CM-H2DCFDA reagent and incubating with 50 μM H_2_O_2_ as described in Materials and methods. Bars represent the H_2_O_2_ permeability of sperm cells expressed as *k* relative. Values are mean ± SEM of two time courses for each of eight different experiments. a, *p* < 0.05 vs. Ctr sub-fertile, HgCl_2_ normospermic, HgCl_2_ sub-fertile (ANOVA, followed by Dunnett *t* test test). Ctr, controls.

H_2_O_2_ permeability of human sperm (and the involvement of AQPs) was measured by a fluorescence method using the CM-H2DCFDA reagent. Two different experimental conditions were used: untreated sperm cells, control and sperm cells treated with 100 μM HgCl_2_ from both normospermic and sub-fertile subjects. The initial rate constant of H_2_O_2_ uptake (*k*) was obtained by setting the time course light scattering with a single exponential equation. H_2_O_2_ permeability of sperm cells from normospermic subjects was about double that of the sub-fertile ones and inhibited by mercury chloride, a known AQP inhibitor (Figure 5). On the contrary, H_2_O_2_ permeability of sperm cells from sub-fertile subjects was unaffected by HgCl_2_ treatment.

Further, sperm cells were treated for 15 min with 100 μM HgCl_2_ and their motility compared with that of untreated (control) cells from both normospermic and sub-fertile subjects. PR motility of sperm from normospermic subjects was significantly decreased by HgCl_2_ treatment, while PR motility of sperm from sub-fertile subjects was unaffected (Table 3).

## 3. Discussion

In this study, we aimed to investigate the involvement of AQPs in the functioning of human sperm cells based on for their properties of either water channels or peroxiporins, which permit osmoregulation (and its related functions) and H_2_O_2_ removal, respectively.

We demonstrated by immunoblotting and immunocytochemistry that multiple AQPs are expressed in human sperm cells: AQP3, -7, -8 and -11 (Figure 1 and Figure 2). Our results confirm previous findings in humans and other species [29,30,31,32,33,34,38,39,40,41].

More complex is the picture about the localization of these AQPs, distributed throughout the sperm cell. Our results suggest that AQP7 is localized in the plasma membrane region of the sperm head (Figure 2). This distribution pattern is similar to that observed by AQP7 staining over the plasma membrane of rat spermatids and spermatozoa [42,43].

Others have reported a localization in the anterior part of the tail [30] or along the tail of human and rat ejaculated sperm [31,32,44]. The absence of AQP7 staining in 20%–60% of sub-fertile and, more interestingly, in 35% normospermic subjects (on the basis of their characteristics in routine sperm analysis) was already demonstrated for both AQP7 and AQP8 by other authors [30,32]. AQP8 showed intense immunoreactivity in the midpiece of the spermatozoa, apparently in the mitochondria and in 2% of sperms in the plasma membrane in the proximity of midpiece, whereas a localization in the tail as granular patches was previously observed [32]. Immunofluorescence experiments evidenced an AQP8 staining restricted to cytoplasmic droplets in rat epididymal spermatozoa [42]. Moreover, the ortholog of human AQP8, named Aqp8b, was found in the midpiece region of seabream sperm [34].

Figure 3 shows that AQP3 immunolabeling is located in the principal piece of the sperm tail membrane and as few granules in the head and in the midpiece. These results are in accordance with a localization in the principal piece of sperm tail of human and rodents as described by Duan and coworkers [29]. Our findings of AQP11 staining in the tail and sometimes in intracellular structures (Figure 3) are similar to previous observations in rodents [33].

As a whole, the pattern of AQPs expression revealed the presence of AQPs in all the portion in human ejaculated sperm cells: in the plasma membrane, in some intracellular structure of the head and the midpiece, and in different portions of the tail. AQP3 functions were previously studied in mice and in humans; the results obtained in AQP3-deficient sperm showed increased vulnerability to hypotonic swelling with increased tail bending occurring after entering the uterus [29]. As a result, sperm motility and fertility are reduced. It was speculated that AQP3 is involved in the regulatory volume decrease.

Although in AQP7-null mice no alteration in the production of sperm or in their morpho- functional properties were observed [45], in humans AQP7 expression was found to be lower in patients than in donors and to be correlated with progressive motility [30,32]. It was suggested that AQP7 may function as a pathway for glycerol entry into sperm cell thus supplying glycerol for energy production [31]. Similarly, AQP8^−/−^ mice showed normal sperm morphology and fertility [38], while human AQP8 seems involved in water transport and in particular in the swelling underlying sperm coiling [32]. Further, no differences between patients and donors were observed.

Finally, it has been speculated that AQP11 may be involved in the end stage of the cytoplasm and organelle elimination process occurring during sperm maturation [33]. More recently, in Syrian hamsters AQP11 was correlated with changes in testis weight and was demonstrated to have a signaling role in the coordinated regulation of crucial components of fertility [41]. As a whole, a functional polarization of the different AQPs in sperm cells was proposed with a role of AQP7 and AQP8 in water influx and a role of AQP3 in water efflux [29].

However, the main goal of this study was to establish the role of AQPs as water and hydrogen peroxide channels and the correlation between their permeability and sperm functioning. In particular we wanted to understand the detoxifying role of AQPs in both normospermic and sub-fertile subjects.

**Figure 6:**
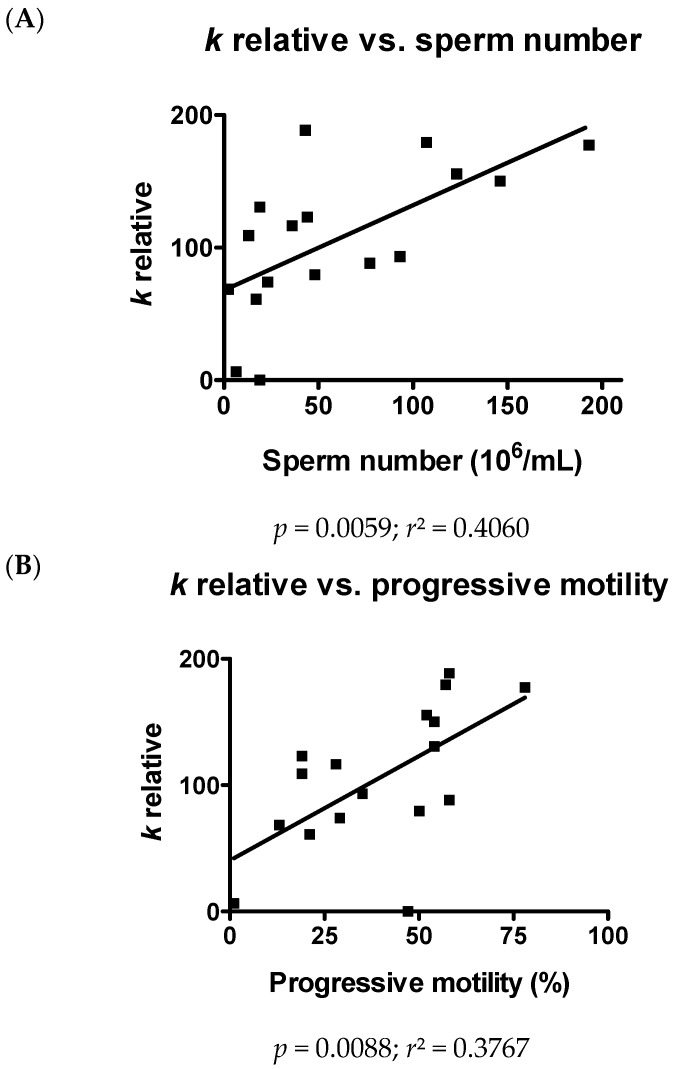
Relationship between osmotic water permeability and sperm number (**A**) or progressive motility (**B**) of human ejaculated semen from all normospermic and sub-fertile subjects. Water permeability of human ejaculated semen was measured by exposure to a 150 mOsm osmotic gradient. Values, expressed as *k* relative, are means of at least 15 single shots. Overall linear regression (black line) is presented. *p* and *r^2^* values are shown.

Water permeability in sperm cells (and the related volume regulation) has been demonstrated one of the greatest of mammalian cells and is of great importance for the linear trajectory of sperm motion and acrosome reaction [46,47,48]. Water permeability of sperm cells here reported was confirmed to be high and to vary from person to person. Further, we have demonstrated that the osmotic water permeability of human ejaculated semen is significantly correlated both with sperm number and progressive motility (Figure 6). Sperm swelling physiologically takes place upon ejaculation when spermatozoa, bathed in the epididymal fluid at about 340 mOsm/L, enter the cervical mucus at a lower osmolarity (about 290 mOsm/L) [46]. The swelling is then followed by fluid exit through the regulatory volume decrease mechanism. However, cell swelling is essential for acrosome reaction and sperm motility [49,50,51]. This suggests a direct involvement of AQPs in sperm concentration and functionality.

The permeability to water cannot be entirely separated from that to H_2_O_2_ and this reflects the ability of some cellular AQPs to promote ROS wasting. The results presented here indicate that various cellular stress conditions, including heat and incubation with H_2_O_2_, reduce water permeability of sperm cells from normospermic subjects. Interestingly, water permeability in sub-fertile subjects is lower than that of the normospermic and is not affected by oxidative stress conditions. Water transport is only partially inhibited by H_2_O_2_ exposure probably because only certain AQPs are permeable to it and therefore are also sensitive to hydrogen peroxide; other water-transporting members of the AQP family in sperm might be H_2_O_2_-insensitive. In this regard, we must consider that only for some AQPs (AQP3, -8 and -9) a permeability to H_2_O_2_ has been demonstrated, while other AQPs, like human AQP1 and AQP4, have a very low transport capacity if any [25,26,27,28,52,53]. In addition, AQP11 reveals a major role in preventing glucose-induced oxidative stress in kidney proximal tubules [54]. To date, among the aquaporins permeable to H_2_O_2_ only AQP8 has been demonstrated to be functionally modulated by H_2_O_2_ [25,52]. Unfortunately, the inability to perform gene silencing of individual AQPs in human sperm does not allow identifying the AQPs responsible for H_2_O_2_ permeation. However, the analysis of functional parameters shows that H_2_O_2_ treatment reduces the vitality of sperm in both normospermic and sub-fertile sperm subjects. The motility is also inhibited by H_2_O_2_ treatment, even if to a different extent, in both normospermic and sub-fertile subjects sperm cells with PR motility affected in normospermic and NP motility in sub-fertile subjects (Table 2).

We demonstrated that AQPs are involved in both water and H_2_O_2_ permeation by showing the inhibitory effect of HgCl_2_, a known water channel inhibitor. Liu et al. did not observed any mercury inhibition [55]; a reduced sperm cells swelling after 25 μM HgCl_2_ addition was reported successively [32]. A correlation between AQP functioning and sperm motility was also observed since a treatment with 50 μM HgCl_2_ reduced almost completely the motility [55]. Our results confirm this finding: mercury treatment inhibited PR motility in normospermic but not in sub-fertile subjects (Table 3). The inhibitory effect of both H_2_O_2_ and HgCl_2_ was reversed by reducing agents. Both water and H_2_O_2_ permeability of sperms from sub-fertile subjects were lower than those from normospermic subjects, with no apparent differences of expression and localization; this may suggest a functional inhibition of the AQPs. Recently, the transport of H_2_O_2_ and of water through plasma membrane AQP8 of HeLa cells was explored [25]. A reduction of H_2_O_2_ and water transport by oxidative stress has been demonstrated, suggesting the existence of a new mechanism that regulates cell signaling and survival during stress [25]. The data here presented seem to support this theory. The chronic deficiency in AQP-mediated H_2_O_2_ permeability may lead to an impaired efflux of ROS from sperm cells, a reduced detoxification mechanism and a loss in sperm functionality.

## 4. Materials and Methods

### 4.1. Sperm Samples

Ninety-one male partners were recruited from infertile couples undergoing infertility evaluation at the Center for Reproductive Medicine, Fondazione IRCCS Policlinico San Matteo (Pavia, Italy) after an informed consent for the processing of semen volume in excess from diagnostic analysis, in accordance to the guidelines approved by our local Ethical Committee on the use of residual biological material for research purpose. Each male produced the semen by masturbation after an abstinence of 2–7 days and semen samples were collected in a sterile plastic bag confirmed to be non-toxic for spermatozoa. A routine semen analysis was performed within 1 h of collection, according to the methods described by the World Health Organization (WHO) [56]. The system used for grading motility distinguishes spermatozoa with PR or NP motility from those that are immotile, as reported by the WHO manual (parameters for normospermic patients: *PR* + *NP* ≥ 40%; *PR* ≥ 32%) [56].

Samples were divided in two groups on the basis of their characteristics: *group* 1–61 samples from subjects defined *normospermic* for the parameters considered with number of spermatozoa ≥ 15 × 10^6^/mL, progressive spermatozoa ≥ 5 mil/mL and physiological viability ≥ 58%); *group* 2–30 samples from patients defined *sub-fertile* with at least one of the principal basal seminal parameters compromised (number of spermatozoa < 15 × 10^6^/mL or *PR* < 32%). In this study, physiological morphology is not considered for discriminating between the two groups patients recruited.

### 4.2. Routine Sperm Analysis

#### 4.2.1. Macroscopic Analysis

Samples were incubated at 37 °C until the analysis was performed. The analysis to assess volume, pH, fluidification and viscosity was started 45 min from semen collection.

#### 4.2.2. Determination of Sperm Count and Motility

Each semen sample was assessed for sperm motility and kinematics of movement using a disposable counting chamber (Counting Chamber Makler, Sefi Medical Instruments, Israel). Sperm count was performed from undiluted specimens. The grid was on a glass cover. The number of spermatozoa counted in any strip of 10 squares of the grid indicated their concentration in millions/mL. No additional factors were necessary for the calculation. We counted at least 3 strips and the median value was considered. The chamber has a depth of 10 microns that eliminates blurring and allows sperm to move freely. The applied sample was observed in one focal plane. The motility of each spermatozoon was graded as follows: PR motility (spermatozoa moving actively); NP motility (all other patterns of motility with an absence of progression); and immotility (no movement) [56].

#### 4.2.3. Determination of Sperm Morphology

To determine sperm morphology, each sample was analyzed by using Diff-Quik-stained slides (Test Simplets, Origio, Denmark). Restricted criteria by Kruger as indicated by the WHO manual were used to analyze at least 200 spermatozoa per sample [56].

#### 4.2.4. Determination of Sperm Viability

Samples were assessed for sperm viability by staining with 1% Eosin-Y in saline (VitalScreen, FertiPro N.V., Belgium). Briefly, 50 μL semen samples were mixed with 2 drops of 1% Eosin-Y in a sterile test tube and a drop of semen-stain mixture was placed on a microscope slide. The smear was covered with a glass cover before the smear was dry and was read immediately under the microscope. At least 200 spermatozoa were counted and classified as stained (dead) or unstained (viable).

### 4.3. Immunoblotting

Human sperm samples were diluted in PBS and centrifuged at 1000× *g* for 15 min. The cell pellets were resuspended with a solution containing: 250 mM sucrose, 1 mM EDTA, 10 mM Tris-HCl, pH 7.6, 0.1 mg/mL PMSF, 100 mM β-mercaptoethanol and Protease Inhibitor Cocktail (P8340, Sigma-Aldrich S.r.l., Milan, Italy) and homogenized by using a Dounce homogenizer. The homogenates were solubilized in Laemmli buffer [57]. Forty micrograms solubilized proteins were subjected to 12.5% SDS-polyacrilamide gel electrophoresis and transferred to the Hybond-P PVDF Membrane (GE Healthcare S.r.l., Milan, Italy) by electroelution. The membranes were incubated overnight with anti-AQP3 rabbit polyclonal IgG (sc-20811, 1:500; Santa Cruz Biotechnology, Inc., Heidelberg, Germany), anti-AQP7 rabbit polyclonal IgG (sc-28625, 1:500; Santa Cruz Biotechnology, Inc.), anti-AQP8 rabbit polyclonal IgG affinity pure (AQP8-A, 1:500; Alpha Diagnostics Intl. Inc., San Antonio, TX, USA), and affinity purified rabbit anti-human AQP11 polyclonal antibody (AP5805b, 1:200; Abgent Inc., San Diego, CA, USA), previously characterized [58,59,60,61]. Control experiments were performed by incubating the blots with pre-immune rabbit serum (not shown). The membranes were washed and incubated for 1 h with goat anti-rabbit IgG antibody, peroxidase conjugated (AP132P; Millipore part of Merck S.p.a., Vimodrone, Italy) diluted 1:100,000 in blocking solution. The bands were detected with ECL™ Select western blotting detection system (GE Healthcare S.r.l., Milan, Italy). Prestained molecular weight markers (ab116028, Abcam, Cambridge, UK) were used to estimate the molecular weight of the bands. Blots were stripped [62] and re-probed with RabMAb anti β-2-microglobulin antibody ((EP2978Y) ab75853; Abcam) as loading control. The antibody was diluted 1:2000 in blocking solution.

### 4.4. Immunocytochemistry

Immunolocalization of AQP3, -7, -8 and -11 was evaluated in human sperm samples as previously described [63]. Samples were smeared on polylysine-coated slides, air dried and fixed in 4% paraformaldehyde in PBS for 30 min, washed with PBS and then treated with 0.3% hydrogen peroxide in methanol for 10 min at room temperature to block the endogenous peroxidases. After washing for 5 min with PBS, sections were blocked with 3% BSA in PBS for 30 min at room temperature. Slides were incubated for 3 h at room temperature with affinity pure primary antibodies (see Immunoblotting sections) diluted 1:200 in antibody diluent (Dako). After three 10 min washes with PBS containing 1% BSA, the sections were first incubated for 30 min at room temperature with biotinylated anti-rabbit IgG and then washed three times with PBS containing 1% BSA for 10 min at room temperature with HRP-conjugated streptavidin (Universal DAKO LSAB^®^ + kit, peroxidase, K0679, DakoCytomation, Milan, Italy). The reaction was visualized by incubation with a DakoCytomation 3,3′-diaminobenzidine chromogen solution. Negative controls were performed by incubating slices with non-immune serum.

The immunostained slides were examined by light microscopy using an Olympus BX41 and the digital images acquired with the Nikon DS-Fi1 digital camera using Nis Element F Imaging Software (2.33, Nikon, Tokyo, Japan).

### 4.5. Water Permeability Measurements

Osmotic water permeability of human sperm samples was measured by stopped-flow light scattering method [64] as previously described [65]. The initial rate constant of sperm cells volume changes (*k*) was obtained by fitting the time course of light scattering with a one phase exponential decay (GraphPad Prism 4.00, 2003, GraphPad Software, Inc., La Jolla, CA, USA).

Water transport was evaluated in: (a) normospermic patients; and (b) sub-fertile patients sperm cells exposed to hypoosmotic solution.

To evaluate the oxidative stress on water permeability, sperm cells were treated with 50 μM hydrogen peroxide (H_2_O_2_) for 45 min at room temperature. To reverse the inhibitory effect of H_2_O_2_, cells treated with H_2_O_2_ were then treated for 15 min with 5 mM DTT. The effect of mercury chloride, a known aquaporins inhibitor, was tested by treating cells for 15 min with 100 μM HgCl_2_ with or without the DTT post treatment. The effect of H_2_O_2_ on routine semen parameters of normorspermic and sub-fertile subjects was also evaluated.

Heat-shock treatment and temperature dependence was performed by placing sperm cells in a water bath set at the desired temperature (37, 38, 39, 40, and 42 °C) for 3 h or left at room temperature (21 °C, Controls).

### 4.6. Hydrogen Peroxide Permeability Measurements

Hydrogen peroxide permeability of human sperm samples was measured by a fluorescence method using the CM-H2DCFDA reagent (Invitrogen, Carlsbad, CA, USA). Briefly, sperm cells were washed in PBS and centrifuged at 1000× *g* for 15 min. The cell pellet was resuspended in PBS and CM-H2DCFDA reagent was added at 5 mM final concentration and incubated for 1 h at room temperature. Thereafter, sperm cells were centrifuged again and the pellet resuspended in PBS. Cells in different experimental conditions (untreated (control) and treated with 100 μM HgCl_2_) were incubated with 50 μM H_2_O_2_; cellular H_2_O_2_ levels were detected over 5 min using a CLARIOstar^®^ microplate reader (BMG LABTECH, Ortenberg, Germany). The initial rate constant of H_2_O_2_ uptake (*k*) was obtained by setting the time course light scattering with a single exponential equation (GraphPad Prism 4.00, 2003).

### 4.7. Protein Content

The protein content was determined with the Bradford method [66] using bovine serum albumin as standard.

### 4.8. Statistics

All data were expressed as mean ± SEM. The significance of the differences of the means was evaluated by using one-way ANOVA followed by Newman–Keuls’s *Q* test or Dunnett *t* test, or Student’s *t* test. All statistical tests were carried out using GraphPad Prism 4.00, 2003.

## 5. Conclusions

**Figure 7:**
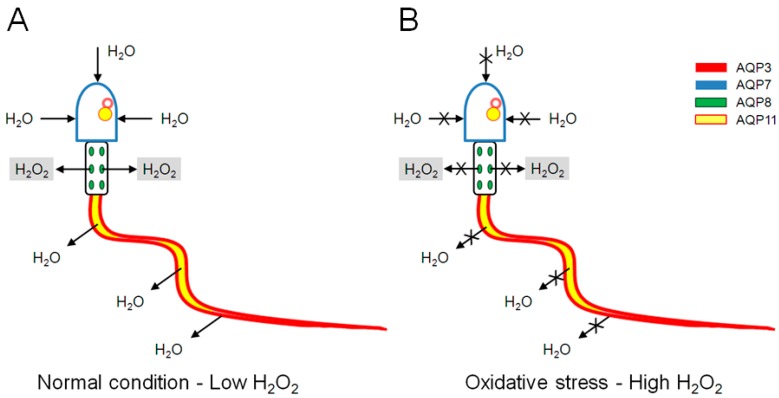
Schematic model of aquaporins (AQP) functioning under normal and oxidative stress conditions: (**A**) under normal condition, water enters through AQP7 and exits through AQP3. H_2_O_2_ produced in low amount exits from mitochondria through AQP8; and (**B**) under oxidative stress condition, H_2_O_2_ reduces water inflow and outflow through AQP7 and AQP3, thus affecting sperm motility. H_2_O_2_ reduces also AQP8 permeability that hampers H_2_O_2_ wasting; this leads to H_2_O_2_ accumulation into mitochondria, reduced AQP production and thus to reduced sperm motility. Intracellular AQP11 could be involved in the end stage of cytoplasm removing.

In conclusion, the present research demonstrated the permeability properties of human sperm AQPs to both water and H_2_O_2_ and the regulatory inhibition role of hydrogen peroxide itself. The chronic impairment in ROS scavenging caused by AQPs inhibition/malfunction can lead to a decrease in sperm number and motility with resulting infertility. A schematic model of AQPs functioning under normal and oxidative stress condition is shown in Figure 7. However, further studies are necessary to understand which AQPs are involved in sperm H_2_O_2_ permeability and the inhibition mechanism.

## Figures and Tables

**Table 1:** Semen parameters of normospermic and sub-fertile patients.

Semen Parameters	Normospermic (*n* = 53)	Sub-Fertile (*n* = 26)
Semen volume (mL)	3.55 ± 0.20	4.22 ± 0.34
Sperm concentration (10^6^/mL)	69.81 * ± 5.58	17.50 ± 4.29
Progressive motility (PR; %)	51.70 * ± 1.76	21.81 ± 2.90
Motile sperm count (10^6^/mL)	37.02 * ± 3.62	3.89 ± 1.05
Non-progressive motility (NP; %)	9.7 ± 0.82	9.15 ± 1.14
Total motility (PR + NP; %)	61.40 * ± 1.78	30.96 ± 3.11
Morphology (% normal)	2.16 * ± 0.26	0.80 ± 0.18

Values are mean ± SEM; * *p* < 0.001 vs. Sub-fertile (Student’s *t* test).

**Table 2:** Effect of hydrogen peroxide on semen parameters of normorspermic and sub-fertile subjects.

Semen Parameters	Normospermic (*n* = 12)		Sub-Fertile (*n* = 12)	
	Before	After	Before	After
Progressive motility (PR; %)	50.17 * ± 3.37	39.92 ± 4.99	22.17 ± 3.18	19.42 ± 3.53
Motile sperm count	45.76 * ± 10.74	35.10 ± 10.12	5.71 ± 1.66	4.76 ± 1.43
Non-progressive motility (NP; %)	6.50 ± 0.91	6.17 ± 0.97	7.08 * ± 1.17	3.33 ± 0.40
Total motility (PR + NP; %)	56.67 * ± 3.61	46.08 ± 4.89	29.25 * ± 3.29	22.75 ± 3.4
Vitality (%)	77.25 * ± 3.83	65.50 ± 5.07	73.5 * ± 2.07	66.42 ± 2.52

Values are mean ± SEM; * indicates *p* < 0.05 compared to parameters after H_2_O_2_ treatment (Student’s *t* test for pair data).

**Table 3:** Effect of mercury chloride (HgCl_2_) on progressive and non-progressive motility of normospermic and sub-fertile subjects.

Semen Parameters	Normospermic (*n* = 8)		Sub-Fertile (*n* = 4)	
	Ctr	HgCl_2_	Ctr	HgCl_2_
Progressive motility (%)	52.5 ± 4.0	43.1 * ± 4.5	32.8 ± 3.7	31.3 ± 3.4
Non-progressive motility (%)	8.1 ± 1.5	6.4 ± 1.0	4.3 ± 1.1	4.0 ± 0.6

Values are mean ± SEM; * indicates *p* < 0.05 compared to control (Ctr) (Student’s *t* test for pair data).

## Abbreviations

ROS	reactive oxygen species
CM-H2DCFDA	5-(and-6)-chloromethyl-2′,7′-dichlorodihydrofluorescein diacetate, acetyl ester
SOD	superoxide dismutase
AQP	aquaporins
FACS	fluorescence-activated cell sorter
WHO	World Health Organization
PR	progressive
NP	non-progressive
DTT	dithiothreitol
